# Impact of Severe Tricuspid Regurgitation on Long Term Survival

**DOI:** 10.5812/cardiovascmed.10686

**Published:** 2013-07-31

**Authors:** Anita Sadeghpour, Mehri Hassanzadeh, Majid Kyavar, Hooman Bakhshandeh, Nasim Naderi, Behshid Ghadrdoost, Arezou Haghighat Talab

**Affiliations:** 1 Echocardiography Research Center, Rajaie Cardiovascular Medical and Research Center, Iran University of Medical Sciences, Tehran, IR Iran; 2 Rajaie Cardiovascular Medical and Research Center, Iran University of Medical Sciences, Tehran, IR Iran

**Keywords:** Tricuspid Regurgitation, Ventricular Function, Right, Ventricular Function, Left

## Abstract

**Background:**

Tricuspid regurgitation (TR) is a common echocardiographic finding, which often accompanies left sided valve disease. Data on mortality and morbidity in patients with severe TR are limited.

**Objectives:**

We sought to assess the outcome of patients with severe TR with the hypothesis that significant TR adversely impacts quality of life and survival, independent of pulmonary artery pressure (PAP) and left ventricular ejection fraction (LVEF).

**Materials and Methods:**

Between 2002 and 2012, 358 consecutive patients (mean age of 54.67± 13.25years, 75.5% female) with severe TR based on history and transthoracic echocardiography (TTE) were enrolled. Patients with severe left sided valvular heart disease and congenital heart disease were excluded. The prevalence of heart failure symptoms, rehospitalization, and duration of hospitalization were evaluated. Survival was calculated according Kaplan Meier curve analysis.

**Results:**

Heart failure (50%) was the most cause of death. Mean years of survival from diagnosis of severe TR was 4.35±3.66, and mean years of survival from onset of symptom was 2.28±1.40. Ninety cases (25.1%) were admitted due to heart failure and through mean of 1.9±0.8 year- follow up (6-32month), 14% of all patients and 36.8% of patients with right heart failure rehospitalized. Atrial fibrillation was reported in 70.5% of patients.

**Conclusions:**

There is a significant increased incidence of mortality, prolonged hospitalization, and rehospitalization in symptomatic patients with severe TR. Therefore, we recommend more aggressive approach toward TV repair or replacement in these patients regardless of PAP and systolic function.

## 1. Background

Tricuspid regurgitation (TR), which often accompanies left sided heart valve disease, is a common echocardiographic finding presents in 80% to 90% of normal individuals (1). TR is mostly functional rather than organic, and is associated with pulmonary hypertension or right ventricular dilatation. Subjective symptoms are often nonspecific, progress very slowly and only become evident after irreversible right ventricular (RV) dysfunction occurs (2). Therefore, determining the optimal time for corrective surgery remains a difficult clinical problem in patients with severe TR (3). Significant residual TR has also been reported in 10% to 45% of patients after TV repair with different techniques (4). Despite the mentioned facts, TR has long been neglected because of the belief that it is a rare and clinically insignificant condition. Patients are rarely referred for isolated surgical tricuspid valve repair, and most repairs are performed in the context of other planned cardiac surgery (4-7). There have been a few studies dealing with this important issue, which have been limited due to small study populations or lack of RV echocardiographic examination (2).

## 2. Objectives

Therefore the aim of this study was to evaluate the incidence of mortality and morbidity associated with severe TR in unmanipulated and in unsuccessful manipulated patients to investigate the hypothesis that significant TR may have adverse effect on survival of the patients, independent of pulmonary artery pressure and left ventricular ejection fraction (LVEF).

## 3. Materials and Methods

Between 2002 and 2012, all patients with valvular heart disease referred to Rajaie Cardiovascular Medical and Research center were evaluated. Patients with significant TR, either surgically manipulated or without any intervention, were enrolled. Exclusion criteria were degree of TR less than moderate to severe, severe left sided valvular heart disease, and significant congenital heart disease.

According to these criteria, 358 patients (with the mean age of 54.67 ± 13.25 years, 14-81 years; 270 female and 88 male cases) were enrolled. Transthoracic echocardiography (TTE) showed additional aortic regurgitation in 237 patients, mitral regurgitation in 292 patients, mitral stenosis in 187 cases, and aortic stenosis in 76 ones. Two cases had pulmonary stenosis (not significant) and 275 patients had pulmonary regurgitation. Other valve involvements were less than moderate. Echocardiography was performed with commercially available ultrasound systems. All patients underwent TTE, and severe TR was defined by echocardiography on the basis of one of the following criteria (8-10): 1.Annulus dilation (4cm or more) or inadequate cusp coaptation, 2. Late systolic flow reversal in the hepatic vein, 3. Regurgitant volume of 45 ml or more, 4. Effective regurgitant orifice (ERO) of 0.4cm 2 or larger, 5. Width of veno contracta of 6.5 mm or more.

The patients underwent a 6- month follow-up in 2 consecutive times with a phone call or personal visit. Clinical course of the patients was also evaluated by documentary review. Proper questionnaire was prepared for data collection, and all the required data obtained from electrocardiogram (ECG), TTE or any diagnostic procedure that performed for the patients during follow up, were also collected and analyzed statistically.

### 3.1. Statistical Analysis

Statistical analyses were performed with SPSS 15 for Windows (SPSS Inc., Chicago, Illinois). Clinical data were expressed as mean values ± standard deviation for interval and count (%) for categorical variables. One sample Kolmogorov-Smirnov test was applied to test the equality of distribution of interval variables with Gaussian distribution. Comparisons between the sub-groups were performed by using Student’s t test (or it’s non[parametric equivalent, Mann Whitney U) test for interval and Pearson’s chi square (or Fisher’s exact) test for categorical variables. Time to events were investigated by Kaplan-Meier method of survival analysis. In all statistical tests, a value of P < 0.05 was considered statistically significant.

## 4. Results

**Figure 1.**
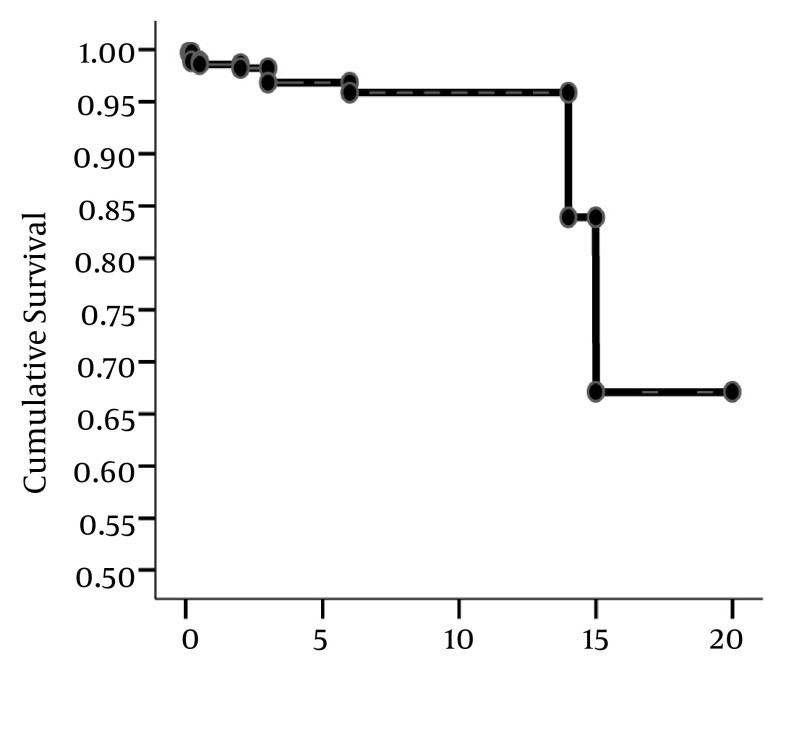
Incidence of Mortality During Follow-Up in Study Group

In this study, 358 patients 270 women (75.5%) and 88 men (24.5%) with moderate to severe or severe TR and the mean age of 54.67 ±13.25 (14-81 years) were participated. The mean year of follow up was 1.9 years (6-32months). Another nonsignificant concomitant valvular involvements were as the following: mitral stenosis in 187 cases (52.2%), mitral regurgitation in 292 cases (81.6%), aortic stenosis in 76 cases (21.2%), and aortic regurgitation in 237 cases (66.2%). 275 cases (76.8%) had pulmonary regurgitation. 81 cases (22.6%) underwent surgical TR repair, and 38.5% experienced significant TR after surgery which were included in the study. Total mortality rate was about 3.3% (12 cases) .The cause of death was right sided heart failure in 6 cases (50%), other causes of death in other 5 cases (41.7%) were as the following: 2 cases of arrhythmia, 2 cases of cardiogenic shock, and 1 case of pulmonary edema. One case of mortality was due to sepsis. The time and probability of mortality has been demonstrated in figure 1 based on Kaplan-Meier method.

Mean time for the diagnosis of severe TR was 4.35 ± 3.66 years, with no significant difference between men and women (P-value = 0.46). Mean time for the incidence of severe TR symptoms was 2.28± 1.40 years with no significant difference between men and women (p-value = 0.39). Based on the results of ECG, 101 cases (28.21%) had sinus rhythm.Ventricular arrhythmia was occurred in 1 patient (0.3%), and atrial arrhythmia was occurred in 257 patients (71.8%), including atrial fibrillation (AF) in 252 patients and flutter rhythm in 5 cases. Thirty-two patients (8.93%) had right-bundle branch block (RBBB) and 7 cases (1.95%) had right ventricular hypertrophy (RVH). The prevalence of left ventricular dysfunction, right ventricular dysfunction, and pulmonary atrial hypertension has been shown in Table 1.

**Table 1.** The Prevalence of Left Ventricular Dysfunction, Right Ventricular Dysfunction, and Pulmonary Atrial Hypertension in Patients with Significant TR

severity	LV^a^. Dysfunction	RV^a^. Dysfunction	PAH^a^
**Normal**	140 (39.1%)	14 (3.9%)	26 (7.3%)
**Mild**	90 (25.1%)	63 (17.5%)	84 (23.4%)
**Moderate**	76 (21.2%)	188 (52.5%)	125 (34.9%)
**Severe**	52 (14.5%)	93 (25.9%)	123 (34.3%)
**Total**	358	358	358

^a^ Abbreviations: TR, tricuspid regurgitation; LV, left ventricle; RV, right ventricle; PAH, pulmonary arterial hypertension

**Figure 2.**
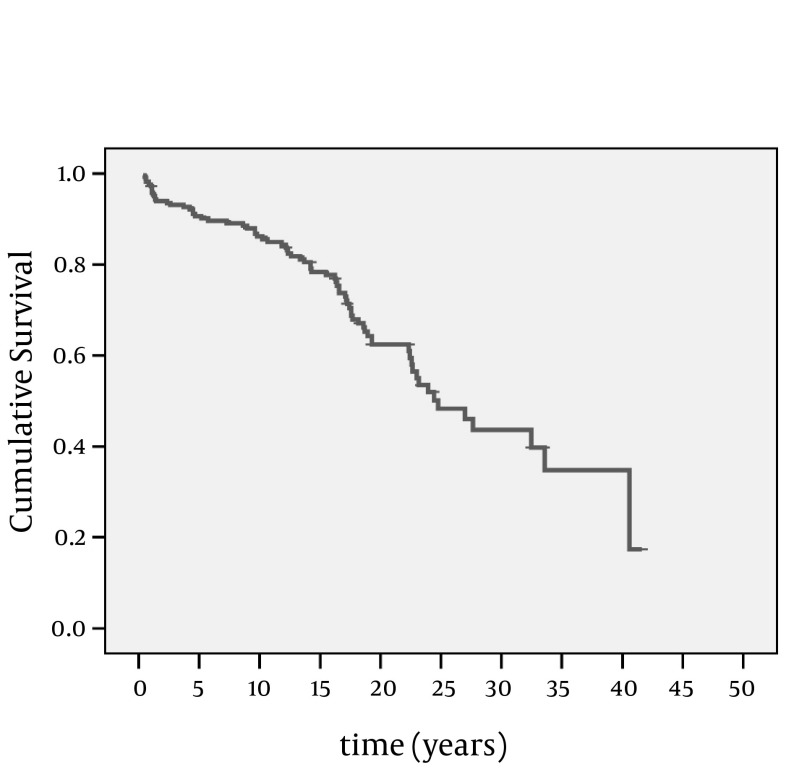
Kaplan Meier Survival Analysis for Heart Failure Diagnosis

Ninety cases with severe TR were admitted to the hospital with no significant difference between men (29.5%) and women (23.6%), (P = 0.26). Fourteen percent of all patients and 36.8% of patients with heart failure were rehospitalized. Seventeen cases (4.7%) were hospitalized for the first time during an average follow up time of 1.9 years (6-32months). Mean interval time between the first admission and heart failure diagnosis was 12.49 ±1.06 years (Figure 2). 

**Figure 3.**
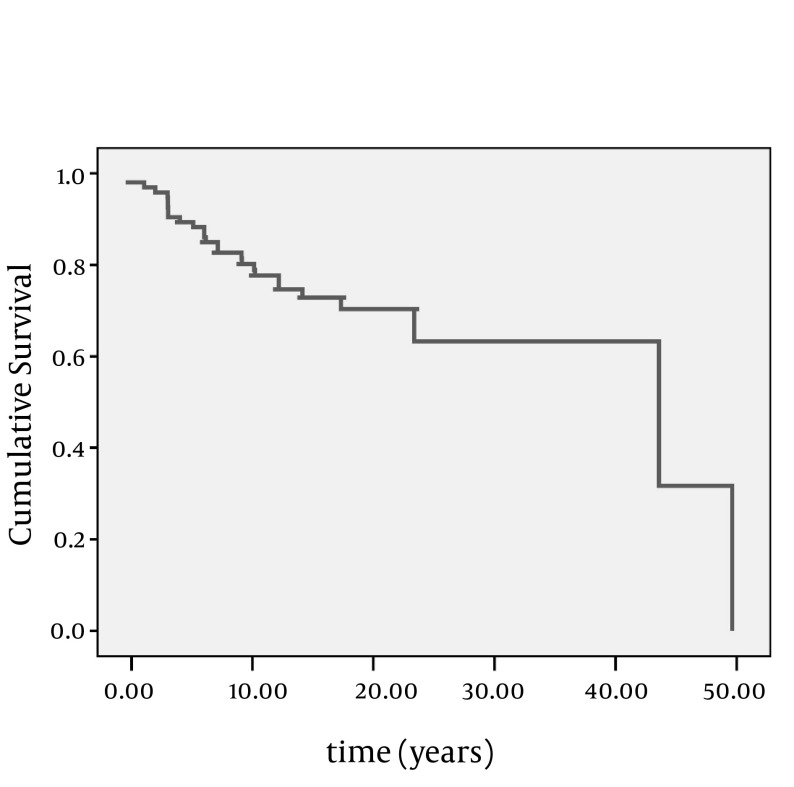
Kaplan Meier Survival Analysis for Rehospitalization

Mean interval time between heart failure diagnosis and rehospitalization was 8.45 ± 2.03 months (Figure 3). 

The incidence of signs and symptoms of heart failure included; dyspnea of exertion (DOE) (66.9%), fatigue (30.1%), edema (26.7%), elevated jugular vein pressure (JVP) (19.5%), palpitation (19.2%), rales (15.4%), ascites (13.1%), and chest pain (10.3%). Overall, 246 cases received diuretic treatment. However, the incidence of DOE functional classes III (41.7%) and IV (5.4%) before diuretic treatment decreased to 26.5% and 0.7%, respectively, after diuretic treatment. The participants were divided based on their pulmonary artery (PA) pressure; primary TR: cases with PA pressure < 55 and secondary TR: cases with PAP ≤ 55. Among total, 225 cases (63%) included in the primary TR group, and 133 cases (37%) in the secondary TR group. These two groups of cases were compared regarding different variables, and the results have been demonstrated in Table 2. 

**Table 2.** Comparison of Different VariablesBetween Patients With Primary and Secondary TR

	Tricuspid Regurgitation		P value
	Primary (n = 226)	Secondary (n = 133)	
**Heart failure sign**			
palpitation	44 (19.5%)	25 (18.8%)	0.876
fatigue	68 (30.1%)	40 (30.1%)	0.998
DOE^a^	141 (62.4%)	99 (74.4%)	0.019
Chest pain	19 (8.4%)	18 (13.5%)	0.126
rales	32 (14.3%)	23 (17.3%)	0.447
edema	59 (26.1%)	37 (27.8%)	0.723
ascites	26 (11.5%)	21 (15.8%)	0.245
Elevated JVP ^a^	43 (19.0%)	27 (20.3%)	0.769
**Arrhythmia**			
atrial	167 (73.9%)	92 (69.2%)	0.335
ventricular	0 (0%)	1 (0.8%)	0.192
**ECG^a^abnormality**			
RBBB^a^	16 (7.1%)	16 (12.0%)	0.112
RVH^a^	3 (1.3%)	4 (3.0%)	0.266
**Death**	9 (4.0%)	3 (2.3%)	0.379
**Cause of death**			0.091
Non cardiac	1 (11.1%)	0 (0%)	
cardiac (RHF^a^)	6 (66.7%)	0 (0%)	
cardiac (none-RHF^a^)	2 (22.2%)	3 (100%)	
**Re-hospitalization**			
Total	27 (12.3%)	22 (16.8%)	0.237
HF	19 (38.0%)	13 (35.1%)	0.784
**Median survival from diagnosis (years)**	4.48 ± 3.63	4.11 ± 3.71	0.128
**Median survival from onset of symptoms (years)**	2.37 ± 1.48	2.16 ± 1.30	0.739

^a^ Abbreviations: DOE, dyspnea of exertion; JVP, Jugular venous pressure; ECG, electrocardiogram; RBBB, right bundle branch block; RVH, right ventricular hypertrophy; RHF, right side heart failure; HF, heart failure; TR, tricuspid regurgitation

Different grades of RV dysfunction, LV dysfunction, and PA hypertension severity were also compared between primary and secondary TR, and the results have been shown in Table 3. 

**Table 3.** Comparison of Different Grades of RV Dysfunction, LV Dysfunction and PA Hypertension Severity Between Patients with Primary and Secondary TR

	Tricuspid Regurgitation		P Value
	Primary(n = 226)	Secondary(n = 133)	
**LV^a^dysfunction**			0.90
Normal	89 (39.4%)	51 (39.1%)	
Mild	56 (24.8%)	34 (25.6%)	
Moderate	51 (22.6%)	25 (18.8%)	
Severe	30 (13.3%)	22 (16.6%)	
**RV^a^dysfunction**			< 0.001
Normal	13 (5.8%)	2 (1.5%)	
Mild	47 (20.8%)	16 (12.0%)	
Moderate	124 (54.9%)	63 (48.1%)	
Severe	42 (18.6%)	51 (38.3%)	
**PAH^a^**			< 0.001
Normal	27 (11.9%)	0 (0%)	
Mild	84 (37.2%)	0 (0%)	
Moderate	104 (46%0)	21 (15.8%)	
Severe	11 (4.8%)	111 (84.2%)	

^a^ Abbreviations: LV, left ventricle; RV, right ventricle; PAH, pulmonary arterial hypertension; TR, tricuspid regurgitation

The patients were also divided into 2 groups based on their gender; 270 female and 88 male cases. Different variables were compared between two groups, and the results have been demonstrated in Table 4. 

**Table 4.** Comparison of Different Variables Between Male and Female Patients

	Gender		P Value
	Male (n = 88)	Female (n = 271)	
**Heart failure sign**			
palpitation	17 (19.3%)	52 (19.2%)	0.979
fatigue	30 (34.1%)	78 (28.8%)	0.345
DOE^a^	57 (64.8%)	183 (67.5%)	0.633
Chest pain	10 (11.4%)	27 (10.0%)	0.715
rales	14 (15.9%)	41 (15.2%)	0.880
edema	31 (35.2%)	65 (24.0%)	0.038
ascites	14 (15.9%)	33 (12.2%)	0.367
Elevated JVP^a^	19 (21.6%)	51 (18.8%)	0.569
**Arrhythmia**			
atrial	58 (65.9%)	201 (74.2%)	0.133
ventricular	0 (0%)	1 (0.4%)	0.568
**ECG^a^abnormality**			
RBBB^a^	6 (6.8%)	26 (9.6%)	0.427
RVH^a^	1 (1.1%)	6 (2.2%)	0.525
**Death**	4 (4.5%)	8 (3.0%)	0.470
**Cause of death**			
Non cardiac	1 (25%)	0 (0%)	
Cardiac (RHF)	0 (0%)	6 (75%)	
Cardiac (none-RHF^a^)	3 (75%)	2 (25%)	0.030
**Re-hospitalization **			
Total	14 (16.5%)	35 (13.2%)	0.443
HF	9 (31.0%)	18 (27.7%)	0.741
**Mean survival from diagnosis (years)**	4.02 ± 3.450	4.45 ± 3.728	0.469
**Mean survival from onset of symptoms (years)**	2.02 ± 1.137	2.37 ± 1.489	0.392
**Diuretic intake**	59 (67.8%)	187 (69.0%)	0.835

^a^ Abbreviations: DOE, dyspnea of exertion; JVP, Jugular venous pressure; ECG, electrocardiogram; RBBB, right bundle branch block; RVH, right ventricular hypertrophy; RHF, right side heart failure; HF, heart failure

Based on the results, there was a significant difference between men and women regarding the severity of TR after surgical repair (p-value: 0.021), furthermore severe TR was significantly more in men than women. There was no significant difference between male and female cases regarding concomitant valvular involvement. However, there was a significant difference between 2 groups regarding heart failure symptoms; therefore, the incidence of edema was significantly more dominant in male than female (p-value:0.038). Different grades of RV dysfunction, LV dysfunction, and PA hypertension severity were also compared between two groups, and there was a significant difference between 2 groups at the base of LV dysfunction (p-value < 0.001). However, there were no significant differences between 2 groups regarding RV dysfunction and PA hypertension severity (p-value: 0.250 and p-value: 0.514, respectively) (Table 5). 

**Table 5.** Comparison of Different Grades of RV Dysfunction, LV Dysfunction, and PA Hypertension Severity Between Male and Female Patients

	Gender		P Value
	Male (n = 88)	Female (n = 271)	
**LV^a^Dysfunction**			< 0.001
Normal	17 (19.3%)	123 (45.8%)	
Mild	20 (22.7%)	70 (25.8%)	
Moderate	24 (27.3%)	52 (19.2%)	
Severe	27 (30.7%)	25 (9.2%)	
**RV^a^Dysfunction**			0.250
Normal	7 (8.0%)	8 (3.0%)	
Mild	13 (14.8%)	50 (18.5%)	
Moderate	38 (43.2%)	149 (55.4%)	
Severe	30 (34.1%)	63 (23.3%0)	
**PAH^a^**			0.514
Normal	10 (11.4%)	17 (6.3%)	
Mild	22 (25.0%)	62 (22.9%)	
Moderate	26 (29.5%)	99 (36.5%)	
Severe	30 (34.1%)	92 (34.4%)	

^a^ Abbreviations: LV, left ventricle; RV, right ventricle; PAH, pulmonary arterial hypertension

## 5. Discussion

Our study showed that significant TR should not be considered benign. Severe TR is associated with higher mortality and hospitalization rates, independent of age, sex, biventricular systolic function and pulmonary hypertension. The incidence of rheumatic fever has been declined in industrialized countries since the 1950s, but in developing countries, it has remained an endemic disease (11). TR has long been neglected because of the belief that it is a rare and clinically insignificant condition. There have been a few studies dealing with this important issue. Therefore, in this study we confirmed that the outcome of patients with significant TR has adverse effect on survival and quality of life of the patients. Surgical management for functional TR can be easily performed; however, the incidence of postoperative morbidity and mortality are high.On the other hand, repair of the tricuspid valve in patients with rheumatic valve disease can be performed with acceptable early results, but progression of rheumatic disease is associated with a high incidence of valvular dysfunction, and mortality in the long term (12). 

Our study demonstrated that the most concomitant valvular disease among our studied group was rheumatic mitral disease with no significant difference between men and women which was similar to other studies. Taramasso M, and Shiran A et al., also concluded in their study that the prevalence of severe TR in patients with MV disease is high. More than 30% of patients with degenerative mitral regurgitation have TR ≥ 2+ at the time of mitral surgery, and up to one-third of patients with significant mitral stenosis have moderate to severe TR (7, 13). Mortality was mostly occurred due to right side heart failure with no significant difference between male and female cases. The patients mostly had atrial fibrillation (70.5%). Similar results were obtained in the study of Kim YJ et al, which 50 cases of 61 patients in their study group (82%) had atrial fibrillation (2). The prevalence of abnormalities in ECG was not significantly different between male and female cases in our study.

Among total, 141 cases had normal LV function, and the cases mostly had moderate RV dysfunction and severe PAH. In the study of Nath J et al, RV dysfunction was reported in 61% of the patients with severe TR (1). The main cause of admission was heart failure in 90 cases. Among total, 14% of cases and among patients with right side heart failure 36.8% of cases were admitted for the second time during a 6 month follow up. 246 cases (68.7%) of our patients received diuretics with significant improvement in their heart failure symptoms. The patients were also divided into 2 groups; primary TR and secondary TR with no significant difference between them. Most of the patients in both primary and secondary TR groups had normal LV function and moderate RV dysfunction. Mild PAH was mostly detected in cases with primary TR; however, the prevalence of severe PAH was significantly more in cases with secondary TR. Patients with severe TR who underwent isolated TV surgery usually have a poor outcome with high perioperative mortality, poor late survival, and no significant improvement in functional capacity in many of them (14-17). Despite guidelines and recent data that support a proactive approach to surgical repair of TR at the time of mitral valve surgery, tricuspid valve repair currently appears underutilized (18). Obviously patients who have severe TR at the time of MV surgery should have their TV repaired at the time of the initial MV surgery and regarding the poor prognosis of untreated patients with severe TR, we would recommend TV repair or replacement before symptoms become overt in these patients. 

There is a significant increased mortality, hospitalization and rehospitalization in symptomatic patients with severe TR. Therefore, more aggressive approach toward TV repair or replacement in these patients regardless of PAP and systolic function, may lead to better outcomes. However, Advising the repair or replacement of the tricuspid valve without the consideration of the right ventricular function and PAP is better to be postponed to the time when suitable interventional studies are performed in this field. This study can be used not only as a documentary but also as a foundation for further studies. 

### 5.1. Study Limitations

The present findings should be interpreted regarding some limitations of the study such as low total mortality rate. Our study was not conducted in a long term follow up, as the mean year of follow up was 1.9 years (6-32months). More studies with larger population and longer follow up are needed.
